# Reviewed article: Bastos ECL, Vidal NAC, Aragão LS, Costa GMAC, Silveira JAC. Marketing violations involving products that compete with breastfeeding in the vicinity of early childhood education centers and health centers: a cross-sectional study based on an audit of the retail food environment, Maceió, 2022-2023. Epidemiol Serv Saude. 2025:34;e20240666.

**DOI:** 10.1590/S2237-96222025v34e20240666.a

**Published:** 2025-09-29

**Authors:** Enilce de Oliveira Fonseca Sally

**Affiliations:** 1 Universidade Federal Fluminense, Niterói, RJ, Brazil Universidade Federal Fluminense Niterói RJ Brazil

## Peer review A

## Questionnaire

Does the manuscript contain new and significant information to justify publication? Yes

Does the Abstract (Summary) clearly and accurately describe the content of the article? Yes

Is the problem significant and concisely stated? Yes

Are the methods described comprehensively? Yes

Are the interpretations and conclusions justified by the results? Yes

Is adequate reference made to other work in the field? Yes

## Manuscript Structure

Length of article is: Adequate

Number of tables is: Adequate

Number of figures is: Adequate

Please state any conflict(s) of interest that you have in relation to the review of this paper. None

Interest: Excellent

Quality: Excellent

Originality: Good

Overall: Good

Recommendation: Accept

## Comments

Seu trabalho possui escrita de fácil compreensão, com linguagem precisa e objetiva e com boa organização das ideias. Sugiro, entretanto que as definições das variáveis referentes às fórmulas infantis sejam revisadas, para que estejam de acordo com a legislação que as regula e com as denominações das marcas presentes em suas embalagens (essa revisão deverá ser feita no tópico Métodos e nas tabelas). Ainda no tópico Métodos, faltou informar que o nome do fabricante do produto constitui uma variável de interesse. No tópico Resultados, convêm revisar alguns valores de frequência de infrações porque não totalizam 100,0% . Quanto às referências, estas são relevantes e apoiam as afirmações feitas no texto, mas senti falta de uma que apoiasse especificamente o resultado acerca da maior frequência de infrações em estabelecimentos pertencentes à rede. Por isso, foi sugerida uma referência para esse conteúdo. Nos comentários do pdf que vai anexo foram feitas sugestões de algumas referências, com vistas a apoiar mais adequadamente resultados apresentados.

